# *Lactobacillus plantarum* Supplementation on Sport Performance, Biomarkers of Intestinal Damage, and Oxidative Stress in Recreational Athletes

**DOI:** 10.3390/jfmk10020131

**Published:** 2025-04-15

**Authors:** Asier Santibañez-Gutierrez, Julen Fernández-Landa, Natalia Busto, Nikola Todorovic, Julio Calleja-González, Juan Mielgo-Ayuso

**Affiliations:** 1 Department of Physical Education and Sports, Faculty of Education and Sport, University of the Basque Country (UPV/EHU), 01007 Vitoria, Spain; santibanez.asg@gmail.com (A.S.-G.); julenfdl@hotmail.com (J.F.-L.); 2 Applied Bioenergetics Lab, Faculty of Sport and Physical Education, University of Novi Sad, 21000 Novi Sad, Serbia; nikolatodorovic1708@gmail.com; 3 Department of Health Sciences, Faculty of Health Sciences, University of Burgos, 09001 Burgos, Spain; nbusto@ubu.es (N.B.); jfmielgo@ubu.es (J.M.-A.)

**Keywords:** *Lactobacillus plantarum*, physical performance, gastrointestinal damage, antioxidant

## Abstract

**Background:** In recent years, interest in probiotic supplementation has increased among athletes due to its potential benefits on sports performance. Thus, the aim of this trial was to investigate *Lactobacillus plantarum*’s effects on sports performance, intestinal damage, and oxidative stress biomarkers. **Methods**: Twenty-two physically active participants, nine females and thirteen males (age: 32.8 ± 5.2 years; height: 1.73 ± 0.1 m (meters); body mass: 72.2 ± 10.3 kg (kilograms) volunteered in this randomized, double-blind, placebo-controlled, parallel study. The participants performed a strenuous exercise session, and immediately after, their perceived exertion was assessed and blood samples were drawn to assess intestinal damage (IFABP: intestinal fatty acid binding protein) and oxidative stress (PC: protein carbonyls; TAC: total antioxidant capacity; total proteins; GSSG: glutathione disulfide; GSH: reduced glutathione and catalase). Twenty-four hours later, the participants ranked their recovery status and completed various sports performance tests: CMJ (countermovement jump), RAST (running-based anaerobic sprint), and YOYO IR1 (YOYO intermittent recovery test level 1). This was followed by a four-week supplementation period, in which the participants ingested one probiotic capsule per day containing 10 billion CFU (colony forming units) of *Lactobacillus plantarum* or a placebo capsule (dextrose). **Results**: The paired samples *t*-test revealed a significantly better result in the YOYO IR1 test in the probiotic group, while a significant reduction was observed in the TAC levels in the placebo group. **Conclusions:** The results suggest that *Lactobacillus plantarum* supplementation could increase YOYO IR1 sports performance test scores and may mitigate TAC value reduction.

## 1. Introduction

Frequent physical activity has several health benefits. However, prolonged and strenuous exercise can stress the gastrointestinal (GI) tract, affecting GI function and decreasing performance [1]. When exercising, blood flow is redistributed to the muscles and organs involved (such as the lungs and heart), while intestinal blood perfusion is reduced, affecting the integrity of intestinal epithelial cells [2]. A disruption of the intestinal tight junctions allows for paracellular absorption, causing the passage of antigens, which leads to inflammation and oxidative stress [3].

Oxidative stress is an imbalance between oxidant and antioxidant levels. Athletes exposed to intense training bouts and, therefore, higher oxygen consumption, may be exposed to higher reactive oxygen species (ROS) production [4,5]. ROS could oxidize proteins, modify their structure, and impair their function, leading to a decrement in muscle force generation during repeated contractions [6,7].

Over the past few years, high-intensity interval training disciplines have become very popular. In this type of training, exercises are repetitively performed at maximal intensity, with minimal or no recovery time [8]. It has been observed that exercising at 70–80% of the maximum oxygen consumption (VO_2max_) decreases splanchnic blood perfusion [9,10]. Moreover, prolonged exercise (≥1 h) and shorter durations (30 min) of resistance training have been demonstrated to increase IFABP circulation, which is a biomarker of enterocyte damage [11]. In this context, dietary strategies, such as the intake of probiotics, could play a key role in mitigating these undesired effects.

The World Health Organization (WHO) has defined probiotics as ‘live microorganisms that, when administered in adequate amounts, confer a health benefit to the host’ [12]. Several mechanisms have been proposed to explain probiotics’ influence on GI functions during exercise, including maintenance of intestinal barrier function, modulation of the immune system due to mucosal adhesion, production of short-chain fatty acids (SCFAs), and enhancement of nutrient absorption [3].

Previous reviews and systematic reviews with meta-analyses have been performed to study the effects of probiotic supplementation on sports performance [3,13,14,15,16,17,18,19,20,21,22]. Nonetheless, the wide range of probiotic strains and the varied supplementation protocols used make it difficult to clarify the effects of probiotics on sports performance and the mechanism of action by which these effects are produced. Furthermore, no study has yet attempted to evaluate the effect of probiotics on intestinal damage and oxidative stress after a strenuous exercise session.

Thus, the principal objective of this trial was to evaluate the effects of 4 weeks of 10 billion CFU of *Lactobacillus plantarum* supplementation on sports performance, a biomarker of intestinal damage and oxidative stress biomarkers in recreational athletes.

## 2. Methods

### 2.1. Participants

The G*Power (version 3.1.9.6; Düsseldorf, Germany) analysis software was used to calculate the optimal sample size, *n* = 28 [23]. Thirty-three (twenty-one males and twelve females) physically active participants from CrossFit^®^ Bikain, Vitoria-Gasteiz (Spain), volunteered in this randomized, double-blind, placebo-controlled, parallel study (age: 34.4 ± 8.1 years; height: 1.7 ± 0.1 m; body mass: 74.6 ± 11.1 kg). Concerning the recruitment procedure, the members of the research group contacted the staff of the training center. The project was explained to members who frequent the training center. The participants voluntarily agreed to participate.

Regarding the eligibility criteria for including participants in this study, the following conditions were considered: age 18 years or older, with a minimum of one year of CrossFit^®^ practice. The exclusion criteria applied included musculoskeletal injuries in the last six months; being diagnosed with a chronic disease; intake of dietary supplements; and the consumption of tobacco, alcohol, medications, antibiotics, and food considered as probiotics (kefir and yoghurt) in the week before the onset of this study. Elite athletes participating in high-level competitions were not included in this study.

The included participants were asked to continue with their training program (three sessions per week) and their usual life habits concerning sleep, hydration, and dietary habits, except for the items mentioned. All participants were informed and given written and oral guidelines regarding the study risks, protocol, and responsibilities.

### 2.2. Study Design

The trial was structured as a randomized, double-blind, placebo-controlled, parallel study. Online computer randomization^®^ was used for the sample randomization (sealedenvelope.com, accessed on 25 November 2023). The trial was registered at clinicaltrials.gov (NCT06220110). The Ethical Committee of the Faculty of Sport and Physical Education of the Basque Country (UPV) provided ethical approval to carry out this intervention (M10/2022/199).

### 2.3. Experimental Procedures

The participants were randomly allocated to two groups: the consumption of one capsule of probiotic containing *Lactobacillus plantarum* (Swanson^®^’s brand; Fargo, ND, USA) 10 billion CFU per day or the consumption of one placebo capsule containing dextrose per day for four weeks. The appearance of both treatments was similar. Participants were asked to take one capsule daily and to continue their usual diet and physical activity habits, except for taking medications or other dietary supplements throughout the intervention. In addition, one week before the initiation of the study, participants were instructed not to consume alcohol, tobacco, medications, probiotics, fermented food, prebiotics, or symbiotics to avoid any interference with the variables measured [24].

Participants completed a 24 h dietary recall on the day they performed the strenuous exercise session and on the following day, when the sports performance measurements were conducted. The aim was to reproduce the diet of these two key days after the 4-week supplementation period. Moreover, to measure GI symptoms, participants answered the Gastrointestinal Symptom Rating Scale (GSRS) questionnaire [25,26].

Body composition assessments were conducted by an internationally certified International Society for the Advancement of Kinanthropometry (ISAK) level 3 anthropometrist. All measurements were performed by the same expert following the ISAK protocol [27]. Height (cm) was recorded with a SECA^®^ measuring rod (Mod. 220; SECA Medical, Bradford, MA, USA), with a 1 mm measuring accuracy. The participants were instructed to stand with their feet together while keeping their heels, buttocks, and upper back in contact with the scale. The head was placed in the Frankfort plane without touching the scale. The measurements were conducted after a deep breath. Body mass (BM) (kg) was evaluated with a SECA^®^ model scale (Mod. 220; SECA Medical, Bradford, MA, USA), with a 0.1 kg accuracy. The participants were weighed in minimal clothing (only their underwear). The scale was zeroed, and then, the participants stood in the center of the scale without support and with their body mass evenly distributed on both feet. Their head was held upright, and their eyes looked straight ahead.

Their subscapular, triceps, abdominal, iliac crest, front thigh, and medial calf muscles were measured using a Harpenden^®^ skinfold caliper with 0.2 mm precision (Harpenden Skinfold Caliper^®^, British Indicators Ltd., London, UK). If the difference between two measurements was over 5% for any skinfold, a third measurement was taken. Their muscle mass and fat percentage were obtained using Lee’s [28] and Carter’s [29] equations, respectively. Girth measurements for their arms, mid-thighs, and calves were recorded using a Lufkin^®^ W606PM (Missouri City, TX, USA) measuring tape (precision: 1 mm).

Before beginning the strenuous exercise session, the participants performed a standardized warm-up [30]. Then, the training session comprised two parts: (I) five sets of barbell back squats, with four repetitions, performed with 75% of their one-repetition maximum (1RM) weight; (II) an as-many-reps-as-possible (AMRAP) circuit of 4 min ON, 1 min REST carried out three times, composed of five devil presses with dumbbells, five thrusters with dumbbells, ten knees to elbows, and ten burpees. The dumbbell weights were self-selected, and the participants were asked to use the same weights in the training session after the supplementation period.

The participants then rated the session’s intensity using a Borg Rating of Perceived Exertion (RPE) scale, ranging from 0 to 10, with 0 being rest and 10 maximal effort [31]. Immediately after, blood samples were collected from the participants’ antecubital veins. The participants ranked their perceived recovery status twenty-four hours after the training session on a scale from 0 (very poorly recovered) to 10 (very well recovered) [32]. Afterward, they performed the same standard warm-up [30] so that three-sport performance tests could be conducted, measuring the following:

1. Power: CMjs were assessed using an optojump (Microgate^®^, Bolzano, Italy). Three vertical jumps were performed with hands on the hips at all times, with a 20 s rest period between jumps.
2. Anaerobic power: The RAST was conducted and scores were recorded with Witty photocells (Microgate^®^, Bolzano, Italy). A total of six 35-m sprints, with a 10 s rest period between each sprint, were carried out. This test measures anaerobic short-distance performance and the time (seconds) a person takes to run each sprint.
3. Aerobic capacity: The YOYO IR1, involving 40 m of distance running (20 m outward and 20 m return) at increasing speeds (starting speed of 10 km/hour, stipulated by an audio beep), was performed. Between each sprint, there were 10 s of active rest (walking in a given area for 5 m). The test ended when the participants did not reach the distance estimated by the beep or when they decided to stop due to fatigue.

**Figure 1:**
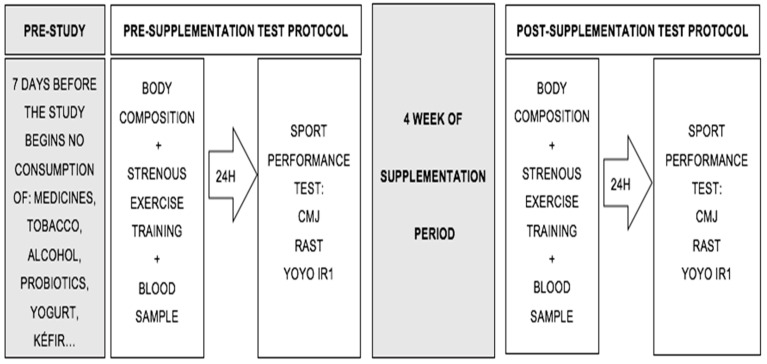
Study design.

After the supplementation period, the participants repeated the same experimental protocol. Figure 1 shows the structure of the study design.

### 2.4. Blood Samples

The same person extracted each blood sample from participants’ antecubital veins immediately after the strenuous exercise session. An ELISA Kit (Hycult Biotechnology^®^, Uden, The Netherlands; detection range: 47–5000 picograms·milliliter^−1^) was used to measure the IFABP concentrations from their plasma.

PCs were assessed following the protocol described by Mesquita CS et al. [33], which is based on the reaction of carbonyl groups with 2,4-dinitrophenylhydrazine to form a 2,4-dinitrophenylhydrazone.

TAC was estimated from plasma iron reducing power (FRAP) values in accordance with the methods described by Harma et al. [34].

The total protein level was determined colorimetrically following the Bradford method. The Bradford reagent was obtained from Sigma Aldrich (Saint Louis, MO, USA). The protein content was estimated following the manufacturer’s instructions.

GSH concentrations were measured based on the reaction of sulphhydryl groups with o-phthalaldehyde (OPT) using a spectrofluorometer. An amount of 10 microliter (μL) of plasma was incubated with 12.5 μL of HPO_3_ (25%) and 37 μL of a phosphate buffer (100 milliMoles (mM), 5 mM EDTA, and pH = 8) at 4 °C for 10 min. Then, the samples were centrifugated for 20 min at 4 °C at 2100× *g*. Subsequently, 10 μL of the supernatant was added to 180 μL of the phosphate buffer (100 mM, 5 mM EDTA, and pH = 8) and 10 μL of OPT (0.1% *w*/*v* in methanol). Then, 15 min after, protected from light and at room temperature, fluorescence was read using a Cytation 5 Cell Imaging multimode reader (Biotek Instruments^®^, Winooski, VT, USA) at λexc = 360 nm and λem = 460 nm. For the GSSG measurements, 10 μL of the undiluted supernatant was reacted for 40 min at room temperature with 4 μL of N-Ethylmaleimide (NEM) to trap GSH. Immediately after, 10 μL of the aforementioned mixture was reacted for 15 min with 10 μL of OPA (0.1% *w*/*v* in methanol) and 180 μL of NaOH 0.1 M. Then, the protocol described for GSH was followed.

For catalase activity, 25 μL of diluted plasma (dilution factor of 20) was mixed with 25 μL of fresh 200 microMol (μM) H_2_O_2_ and incubated for 30 min at room temperature. Then, 50 μL of Amplex Red reagent (100 μM Amplex Red; 4 U/mL HRP: horseradish peroxidase in 100 mM Tris-HCl pH = 7.5) was added to measure the catalase activity (AR-CAT) in each well by a multichannel micropipette. Subsequently, the plate was incubated for 30 min at 37 °C. Finally, after moderate shaking for 3 s in the plate reader, the fluorescence signal (λ excitation = 530 ± 25 nm and λ emission = 590 ± 25 nm) was read using a Cytation 5 Cell Imaging multimode reader (Biotek Instruments^®^, Winooski, VT, USA).

### 2.5. Statistical Analysis

To measure the variables’ normality and homoscedasticity, the Shapiro–Wilk normality test (*n* < 30) and Levene’s test were conducted, respectively. All data are provided as mean ± standard deviation (SD). A two-way mixed design ANOVA was applied to evaluate the effects of group, time, and group × time. The effect sizes were calculated as partial η^2^ (η^2^p), with thresholds of small (0.01–0.059), medium (0.060–0.139), and large (>0.140), per Ferguson’s guidelines [35]. Additionally, paired and independent *t*-tests were conducted to measure the differences within and between groups. Their equivalents, a non-parametric analysis, the Mann–Whitney U test (for between-group differences), and the Wilcoxon signed-rank test (for within-group differences), were applied when the data were not normally distributed. The effect size was interpreted as trivial when it was <0.2, small if 0.2–0.3, moderate if 0.4–0.8, and large if >0.8, according to Cohen’s criteria [36]. Significance was set at *p* < 0.05. A Microsoft Excel^®^ spreadsheet (Microsoft Corporation, Redmond, Washington, DC, USA) was used to register all data. For the analysis, Statistical Package for Social Sciences software version 26.0 (SPSS^®^ Inc., Chicago, IL, USA) was used.

## 3. Results

Eleven participants did not succeed in completing the whole process because of muscle issues or the impossibility of coming to the facilities on the required days. Five participants (four males and one female) belonged to the probiotic group, and six people (four males and two females) formed part of the placebo group. Finally, twenty-two participants completed the whole trial. Although this figure is lower than initially determined, the rigorous methodology applied makes it a scientifically solid study. The placebo group consisted of 10 participants (6 males and 4 females), while the probiotic group comprised 12 participants (7 males and 5 females). Concerning the analysis for paired samples, non-parametric statistics were applied for body mass, body mass index (BMI), recovery scale, GSSG, and catalase in the probiotic group and for body mass and YOYO IR1 in the placebo group.

The physical characteristics of the placebo and probiotic groups were similar at baseline. No statistical differences between groups were found in age, height, body mass, or BMI. For further information, see Table 1.

Regarding body composition, none of the treatments produced significant differences. For additional information, see Table 2.

In regard to sports performance tests, RPE, recovery scale, and GI survey, the 2-way mixed-design ANOVA showed no differences between treatments. However, paired Student’s *t*-tests showed a significant increment in YOYO IR1 test scores in the probiotic group (Pre-Probiotic: 883.3 ± 436.7 vs. Post-Probiotic: 1066.7 ± 502.9; *p* < 0.05). Further information is detailed in Table 3.

Concerning the blood biomarkers analyzed, Student’s *t*-test for independent samples were applied regarding TAC levels, noting significant differences between groups in the pre-supplementation period (Pre-Placebo: 1108.2 ± 518.8 vs. Pre-Probiotic: 975.0 ± 332.0; *p* < 0.05). In addition, when paired samples Student’s *t*-tests were conducted, a significant reduction in TAC levels was reported in the placebo group (Pre-Placebo: 1108.2 ± 518.8 vs. Post-Placebo: 809.0 ± 374.7; *p* < 0.05). Detailed information is displayed in Table 4.

## 4. Discussion

This trial was performed to determine the effects of *Lactobacillus plantarum* supplementation on sports performance, intestinal damage, and oxidative stress biomarkers. None of the included participants reported any side effects after the intake of this supplement.

The results showed that 10 billion CFU of *Lactobacillus plantarum* positively affected the YOYO IR1 test in the within-group analysis conducted for the probiotic group. In addition, the within-group analysis of TAC levels showed a significant decrease in the placebo group, while the values of the probiotic group were not significantly reduced. However, the lack of specific information regarding the particular strain of *Lactobacillus plantarum* used adds uncertainty that must be considered.

Despite the lack of significant differences in this study in relation to body composition, probiotics, through SCFA production and microbiota modification, may be involved in certain mechanisms of action that affect weight reduction. Intestinal microorganisms can transform complex nutrients, such as fibers and non-digestible carbohydrates, into simple sugars and, through fermentation, produce SCFAs (acetate, propionate, and butyrate) [37]. SCFAs may activate fatty acid oxidation and inhibit de novo synthesis and lipolysis. Consequently, body mass may be reduced [38]. However, in this trial, no differences were found in body fat and muscle mass percentage, a finding which is in accordance with the results observed in other studies using *Lactobacillus plantarum* TWK10 [24,39,40].

In regard to sports performance, a previous systematic review and meta-analysis assessing probiotic effects on strength and power showed significantly greater results after probiotic supplementation [21]. The only trial reporting significant positive results was performed using *Lactobacillus plantarum* PS128 [40]. In that trial, the authors investigated the effects of probiotic supplementation after athletes completed an official triathlon competition to assess the benefits of probiotics on performance and recovery. Thus, their post-supplementation period measurements were performed after the triathlon competition. A Wingate test was carried out 72 h after the triathlon to measure anaerobic capacity. The results revealed significant improvements in the probiotic group’s mean power. Another study explored lower extremity explosive strength, measured by a CMJ test, before and after running a half marathon. The results revealed that the placebo group significantly reduced lower-limb explosive strength 0, 3, and 24 h after the half marathon, while the probiotic group was able to maintain their lower-limb explosive strength [41].

Moreover, in the study performed by Huang et al. [40], an endurance performance test, running at 85% VO_2max_ until exhaustion, was conducted 48 h after a triathlon. The probiotic group could maintain their performance between pre- and post-supplementation. The authors suggested that these results were likely due to the ability of this strain to modulate exercise-induced inflammation and oxidation. Their results are in accordance with the findings observed in this trial, where significantly better results were reported in the YOYO IR1 test 24 h after the strenuous exercise session in the probiotic group. Nevertheless, no improvements were noticed in the CMJ and RAST tests.

Although SCFA concentration was not measured in this study, a previous trial revealed increased SCFA concentration after supplementation with *Lactobacillus plantarum* PS128 [39]. Acetate, the SCFA found in the highest concentrations in the bloodstream, may be converted into acetyl-CoA in peripheral muscles for oxidation or lipogenesis [42]. Propionate is involved in liver gluconeogenesis [43,44,45] and in protecting the intestinal barrier [42]. The colonocytes consume most of the butyrate [46]. Butyrate plays a key role in regulating intestinal tight junction proteins and increasing intestinal barrier function [47]. This could explain the notably lower values found (although not significant) in the probiotic group concerning the marker of intestinal damage. Although no significant differences were observed in the survey related to GI problems, probiotic groups showed better values than placebo groups in all the applied analyses.

Furthermore, by improving intestinal barrier function, probiotics could reduce the endotoxins produced by exercise and the associated cytokine production [48]. Moreover, the inflammation produced by intense exercise increases free radical activity [49]. By increasing SCFA production, probiotics may inhibit the production of pro-inflammatory cytokines and up-regulate antioxidant enzymes to improve inflammation and oxidative stress status [50]. In addition, probiotics may be involved in releasing antioxidant molecules, secreting antioxidant enzymes [51], and improving the absorption of antioxidants and thus their availability [52,53]. In a study conducted by Fu et al. [41], 74 and 96 h after a half marathon, antioxidant function was significantly higher in the probiotic group concerning the superoxide dismutase (SOD) biomarker, but no significant differences were observed in CAT. Probiotics’ effects on improving exercise capacity were related to probiotics’ anti-inflammatory and antioxidant effects. A systematic review and meta-analysis conducted by Zamani et al. observed a significant reduction in malondialdehyde (MDA) and a significant increase in TAC level. In contrast, no differences were obtained in GSH levels [54]. In the present study, probiotic supplementation prevented a significant reduction in TAC values, which is a biomarker of the body’s redox state, suggesting that it may assist in mitigating the oxidative stress that results from high-intensity exercise. Nonetheless, no significant differences were observed between treatments in the other measured blood parameters.

This study is not exempt from certain limitations, meaning the observed results should be cautiously interpreted. For instance, the study’s sample size (*n* = 22) can be considered small. Moreover, diet was not entirely controlled during this project, although dietary intake was recorded on the most critical days. Another limiting factor is the lack of analysis of other markers, such as SCFA from feces or pro-inflammatory cytokines, which would contribute to understanding probiotics’ mechanism of action. In addition, the uncertainty associated with the specific probiotic strain used in this research limits the possibility of extrapolating the results found in this study. Future studies are essential to confirm the results observed in the current study and to fully understand the underlying mechanisms by which probiotics influence athletic performance. In addition, further research is needed to determine whether the effects of probiotics may vary between genders with the aim of providing a better understanding of their potential benefits in different populations.

## 5. Conclusions

The administration of 4 weeks of 10 billion CFU of *Lactobacillus plantarum* benefits participants in the YOYO IR1 test and prevents TAC levels from reducing. Nevertheless, further studies are crucial to clarify the mechanisms of action by which probiotics affect sports performance and to establish a solid scientific basis for probiotic supplementation in the sports field.

## Figures and Tables

**Table 1:** Descriptive characteristics of the placebo and probiotic groups at baseline.

Variable	Placebo Group (*n* = 10) Mean ± SD	Probiotic Group (*n* = 12) Mean ± SD
Age (years)	32 ± 5	33 ± 5
Height (m)	1.7 ± 0.1	1.7 ± 0.1
Body mass (kg)	71.8 ± 10.3	72.4 ± 10.8
BMI (kg/m^2^)	24.1 ± 2.1	24.1 ± 2.0

Note. m^2^, square meters; *n*, number of participants; SD, standard deviation.

**Table 2:** Results of Student’s *t*-test for paired samples and the Wilcoxon signed-rank test that measured within-group differences between the pre- and post-test measurements on body composition.

					95% CI for Effect Size	
Variables	Pre (Mean ± SD)	Post (Mean ± SD)	*p*	Effect Size	Lower	Upper
Body mass (kg)						
Placebo (*n* = 10)	71.9 ± 10.3	72.2 ± 9.9	0.906	0.236	0.735	0.429
Probiotic (*n* = 12)	72.4 ± 10.8	72.9 ± 10.8	1.000	−0.410	0.792	0.202
BMI (kg/m^2^)						
Placebo (*n* = 10)	24.1 ± 2.1	24.1 ± 2.3	0.904	0.039	0.582	0.658
Probiotic (*n* = 12)	24.1 ± 2.0	24.3 ± 1.9	0.526	0.410	0.792	0.202
BF%						
Placebo (*n* = 10)	12.2 ± 3.4	12.2 ± 3.6	0.873	0.052	0.671	0.570
Probiotic (*n* = 12)	14.5 ± 3.8	14.4 ± 3.7	0.445	0.229	0.350	0.798
Muscle mass%						
Placebo (*n* = 10)	41.7 ± 3.1	42.0 ± 2.9	0.530	0.206	0.828	0.426
Probiotic (*n* = 12)	39.3 ± 5.2	39.4 ± 4.7	0.879	0.045	0.610	0.522

Results expressed as mean ± standard deviation. Note. BF, body fat; CI, confidence interval; %, percentage.

**Table 3:** Results of Student’s *t*-test for paired samples and the Wilcoxon signed-rank test regarding sports performance, RPE, recovery scale, and GI survey.

					95% CI for Effect Size	
Variables	Pre (Mean ± SD)	Post (Mean ± SD)	*p*	Effect Size	Lower	Upper
CMJ (cm)						
Placebo (*n* = 10)	32.5 ± 7.4	32.4 ± 7.4	0.864	0.056	0.566	0.674
Probiotic (*n* = 12)	33.3 ± 8.5	33.9 ± 8.9	0.085	0.546	1.144	0.074
RAST (s)						
Placebo (*n* = 10)	6.1 ± 0.6	6.1 ± 0.6	0.693	0.129	0.497	0.748
Probiotic (*n* = 12)	6.2 ± 0.7	6.2 ± 0.6	0.970	0.011	0.555	0.577
YOYO IR1 (m)						
Placebo (*n* = 10)	1080.0 ± 432.9	1200.0 ± 545.2	0.083	0.636	0.896	0.053
Probiotic (*n* = 12)	883.3 ± 436.7	1066.7 ± 502.9	0.003 *	1.083	1.789	0.347
RPE						
Placebo (*n* = 10)	6.7 ± 2.1	7.6 ± 0.8	0.171	0.470	1.115	0.197
Probiotic (*n* = 12)	6.7 ± 2.1	7.4 ± 0.5	0.564	0.172	0.738	0.402
Recovery scale						
Placebo (*n* = 10)	7.7 ± 1.5	6.6 ± 2.6	0.116	0.551	0.131	1.206
Probiotic (*n* = 12)	7.1 ± 1.9	6.6 ± 1.8	0.551	0.538	0.846	0.039
GI survey						
Placebo (*n* = 10)	24.3 ± 7.7	27.4 ± 9.9	0.304	0.345	0.303	0.975
Probiotic (*n* = 12)	26.7 ± 11.4	23.7 ± 10.1	0.286	0.324	0.898	0.264

Results reported as mean ± standard deviation. Note. s, seconds; * indicates statistical significance (*p* < 0.05).

**Table 4:** Student’s *t*-test for paired samples and Wilcoxon signed-rank results concerning measured blood biomarkers.

					95% CI for Effect Size	
Variables	Pre (Mean ± SD)	Post (Mean ± SD)	*p*	Effect Size	Lower	Upper
IFABP						
Placebo (*n* = 10)	565.1 ± 305.7	497.1 ± 393.3	0.587	0.178	0.452	0.798
Probiotic (*n* = 12)	680.0 ± 321.1	535.9 ± 369.8	0.268	0.337	0.253	0.912
PC						
Placebo (*n* = 10)	0.9 ± 0.3	0.9 ± 0.3	0.455	0.247	0.870	0.390
Probiotic (*n* = 12)	0.9 ± 0.2	1.00 ± 0.16	0.465	0.219	0.787	0.359
TAC						
Placebo (*n* = 10)	1108.2 ± 518.8	809.0 ± 374.7	0.048 *	0.723	0.006	1.409
Probiotic (*n* = 12)	975.0 ± 332.0	696.1 ± 423.7	0.098	0.523	0.093	1.118
Total proteins						
Placebo (*n* = 10)	7.2 ± 0.9	7.5 ± 0.7	0.460	0.244	0.867	0.392
Probiotic (*n* = 12)	7.5 ± 1.0	7.1 ± 0.9	0.419	0.243	0.337	0.812
GSSG						
Placebo (*n* = 10)	374.3 ± 130.3	287.4 ± 131.9	0.125	0.536	0.143	1.189
Probiotic (*n* = 12)	319.1 ± 106.9	333.9 ± 139.9	0.754	0.538	0.658	0.477
GSH						
Placebo (*n* = 10)	1337 ± 351.1	1254 ± 340.3	0.586	0.179	0.451	0.799
Probiotic (*n* = 12)	1206 ± 374.9	1105 ± 261.6	0.460	0.221	0.357	0.789
Catalase						
Placebo (*n* = 10)	0.03 ± 0.023	0.04 ± 0.33	0.131	0.526	1.178	0.151
Probiotic (*n* = 12)	0.04 ± 0.04	0.06 ± 0.11	0.791	0.103	0.491	0.631

Results illustrated as mean ± standard deviation. Note. IFABP is expressed as picograms/milliliter; PC is expressed as micromol/milligram protein; TAC is reported as Trolox Equivalents (microM); total proteins are reported as gram/deciliter; GSSG is expressed as microgram of GSSG/microgram of protein; GSH, is expressed as microgram of GSH/microgram of protein; catalase is reported as Unit/millimeter; * indicates statistical significance (*p* < 0.05).

## Data Availability

The data presented in this trial are available in this article.
